# Cardiac troponin and cerebral herniation in acute intracerebral hemorrhage

**DOI:** 10.1002/brb3.697

**Published:** 2017-04-21

**Authors:** Mangmang Xu, Jing Lin, Deren Wang, Ming Liu, Zilong Hao, Chunyan Lei

**Affiliations:** ^1^ Department of NeurologyWest China HospitalSichuan UniversityChengduChina; ^2^ Center of Cerebrovascular DiseasesWest China HospitalSichuan UniversityChengduChina; ^3^ Department of NeurologyFirst Affiliated Hospital of Kunming Medical UniversityKunmingChina

**Keywords:** biomarker, cardiac troponin, cerebral herniation, intracerebral hemorrhage

## Abstract

**Objectives:**

To explore the association, if any, between the relationship between cardiac troponin and cerebral herniation after intracerebral hemorrhage (ICH).

**Methods:**

Six hundred and eighty‐seven consecutive ICH patients admitted to West China Hospital from May 1, 2014 to September 1, 2015 were retrospectively reviewed. Data on demographics, etiology, laboratory examinations at admission including serum cardiac troponin, computed tomography (CT) scans at admission and follow‐up, and clinical outcomes were obtained. Using multiple logistic regression to identify the relationship of troponin and herniation. The association between troponin and hematoma volume was assessed using bivariate correlation and linear regression.

**Results:**

Among 188 (27.4%) patients who underwent the test of serum cardiac troponin at admission, 16 (8.5%) demonstrated cerebral herniation. The median time from symptom onset to CT at admission and follow‐up was 4 and 30.25 hr, respectively. In multivariate analysis, elevated troponin was independently associated with cerebral herniation (adjusted odds ratio [OR] 5.19; 95% confidence interval [CI], 1.08–24.93). And those with elevated troponin had larger hematoma volume at follow‐up in bivariate correlation (correlation coefficient, .375, *p* = .003) and linear regression (β, .370, 95% CI, 0.062–0.320, *p* = .005), higher National Institutes of Health Stroke Scale score (adjusted OR 2.06; 95% CI, 1.06–4.01, *p* = .033) and lower Glasgow Coma Scale score (adjusted OR 2.34; 95% CI, 1.17–4.68, *p* = .016) than those without.

**Conclusions:**

Elevated cardiac troponin was associated with an almost five‐fold increased risk of cerebral herniation, but not in‐hospital mortality. The possibility of cerebral herniation should be considered when ICH patients with large hematoma volume and elevated troponin.

## 1 Introduction

Cerebral herniation is a severe complication after intracerebral hemorrhage (ICH) which often results in the dysfunction of brain stem and cranial nerves (Gower, Baker, Bell, & Ball, 1987; Koenig et al., 2008) and requires immediate diagnosis and neurological life support (Stevens, Shoykhet, & Cadena, 2015). The recognition of cerebral herniation is very important due to its high mortality.

On the other hand, serum cardiac troponin is closely associated with vascular events (Everett, Zeller, Glynn, Ridker, & Blankenberg, 2015) and has been widely used as the biomarker for myocardial infarction and cardiac damage after stroke (Naidech et al., 2005). Recently, elevated troponin has been found to be associated with poor outcome and mortality in stroke patients (Batal et al., 2016; Raza & Alkhouli, 2014; Thalin et al., 2015). Thus, serum troponin levels might play a pivotal role in stroke patients and are associated with the severity of stroke. However, most of the studies on cardiac troponin and stroke were performed in patients with cerebral infarct or subarachnoid hemorrhage (SAH; Batal et al., 2016; Zhang, Wang, & Qi, 2015). Little is known about the effect of cardiac troponin in ICH patients, especially the relationship of elevated troponin and cerebral herniation, as well as the association between elevated troponin and hematoma volume.

Thus, we aimed to explore the association, if any, between serum troponin and cerebral herniation, as well as hematoma volume after ICH.

## 2 Methods

### 2.1 Patients

Within the Scientific Research Department of West China Hospital approval, we retrospectively reviewed ICH patients who had the test of serum cardiac troponin at admission. We identified 687 consecutive computed tomography (CT)‐positive ICH patients admitted to West China Hospital from May 1, 2014 to September 1, 2015. Traumatic ICH, primary SAH and Hemorrhagic transformation of cerebral infarct were excluded. Among the 687 ICH patients, 188 (27.4%) had the test of serum cardiac troponin at admission. The methodology of our study was in accordance with local ethics criteria for human research. All patients or their guardians provided informed consent for participation.

### 2.2 Data collection

We derived data on demographics, doubtful risk factors for ICH, data of electrocardiogram, clinical characteristics and time to CT from the prospective database of the National Key Technology R&D Programme of the 12th Five‐Year Plan which had been reported previously (Lei et al., 2015). The severity on admission was measured using the Glasgow Coma Scale (GCS) and the National Institutes of Health Stroke Scale (NIHSS; Brott et al., 1989) by certified physicians.

The biomarkers related to coagulation function, renal function, cardiac biomarkers including cardiac troponin, myoglobin, and creatine kinase‐MB mass (CK‐MB), and serum ions. Blood samples were taken and processed as soon as possible while at admission. Imaging information (cerebral herniation, hematoma volume, the location of hematoma, presence of intraventricular hemorrhage, combined with SAH) is limited to CT scans or CTA. Etiology of ICH was categorized as hypertension, aneurysm, arteriovenous malformation, moyamoya disease, severe systemic disease, or unknown. Data on in‐hospital mortality was also collected. Available CT scans were independently reviewed by M.X. and J.L.

### 2.3 Definitions

On CT scans, cerebral herniation was characterized by uncontrollable diffuse brain swelling or mass effect resulted from predominantly unilateral brain swelling, often combined with obliteration of perimesencephalic cisterns (Bor‐Seng‐Shu et al., 2013). Hematoma volume was calculated using the formula of *ABC*/2 where *A* is the maximum diameter of the greatest cross section of the hematoma, *B* is perpendicular to *A* and *C* represents the slice thickness multiplied by the number of CT slices (Kothari et al., 1996). The volume of hemorrhage flooding into ventricular was not included into the measurement of hematoma volume. The location of hematoma was categorized into deep, lobe, posterior fossa, primary intraventricular hemorrhage and multilple/undefined. Large hematoma volume was defined as over 30 ml. Elevated troponin was defined as over 14 ng/L. Elevated myoglobin was defined as over 58 ng/ml. Elevated CK‐MB was defined as over 4.94 ng/ml. Using the Formula of Modification of Diet in renal disease to calculate estimated glomerular filtration rate (eGFR): eGFR = 186 × (Scr)^−1.154^ × (age)^−0.203^ × (0.742 if female; National Kidney Foundation, 2002). Chronic kidney disease (CKD) was defined as eGFR < 60 ml/min per 1.73 m^2^. End stage renal disease (ESRD) was defined as eGFR < 15 ml/min per 1.73 m^2^.

### 2.4 Statistics

Baseline characteristics were compared using the χ^2^ test or fisher exact test for categorical data, and Student *t* test or Mann–Whitney *U* test for continuous data, as appropriate. Using multivariate logistic regression to study the association between elevated serum troponin and the incidence of cerebral herniation. The association between troponin and hematoma volume was assessed using bivariate correlation and linear regression. In the linear regression, we adjusted for age, GCS score and systolic blood pressure at admission.

## 3 Results

During the study period, a total of 188 (27.4%) patients were enrolled. Among all these 188 subjects with complete data, mean age was 56.34 years. 60.6% were men, and 66 (35.1%) had elevated serum cardiac troponin. The median time from symptom onset to CT at admission and follow‐up was 4 and 30.25 hr, respectively. The mean hematoma volume was 30.04 ml (median, 24.34, range, 32.05) and 39.9% were originated in deep.

Among these 66 patients with elevated troponin, one had cardiac ischemia characterized by atrial premature beats, the change of T‐wave and left anterior fascicular block on electrocardiogram; other electrocardiogram abnormalities included atrial fibrillation in one patient and supraventricular tachycardia in one patient. In addition, 5 of the 66 patients had pre‐existing heart disease which included coronary heart disease, myocardial infarction and congenital heart disease.

Table 1 showed the baseline clinical characteristics between patients with and without cerebral herniation. Most of the characteristics were comparable, but subjects with cerebral herniation were more likely to have lower GCS score (*p* = .003), higher NIHSS score (*p* = .003), higher proportion of elevated serum troponin (*p* = .016) and larger hematoma volume (*p* = .002) compared with patients without cerebral herniation.

**Table 1:** Baseline characteristics of the study population and the association between elevated troponin and cerebral herniation

Characteristic	Total	Non‐cerebral hernia (*n* = 172)	Cerebral hernia (*n* = 16)	*p* Value
Age, mean (*SD*)	56.34 (16.79)	56.31 (16.85)	56.56 (16.67)	.955
Male, %	114 (60.6)	101 (58.7)	13 (81.3)	.078
Hypertension, %	87 (46.3)	78 (45.3)	9 (56.3)	.403
Diabetes mellitus, %	16 (8.5)	15 (8.7)	1 (6.3)	1.000
Cardiac events, %	14 (7.4)	13 (7.6)	1 (6.3)	1.000
Previous stroke, %	21 (11.2)	20 (11.6)	1 (6.3)	.812
Alcohol intake, %	29 (15.4)	28 (16.3)	1 (6.3)	.484
Smoking, %	49 (26.1)	45 (26.2)	4 (25)	1.000
Systolic blood pressure, mean (*SD*)	160.29 (34.02)	159.16 (34.21)	172.25 (30.36)	.142
Diastolic blood pressure, mean (*SD*)	94.68 (20.93)	94.24 (20.93)	99.25 (21.05)	.362
GCS, median (IQR)	12 (9)	12 (8)	6 (7.5)	.003
NIHSS, median (IQR)	10 (18.5)	10 (15.75)	27.5 (19.25)	.003
Coagulation function				
PT, mean (*SD*)	13.07 (9.17)	13.17 (9.55)	11.99 (1.06)	.658
APTT, mean (*SD*)	27.73 (6.84)	27.81 (7.02)	26.80 (4.31)	.611
INR, mean (*SD*)	1.11 (0.43)	1.11 (0.45)	1.07 (0.09)	.763
Fibrinogen, mean (*SD*)	3.07 (0.97)	3.09 (0.97)	2.88 (0.98)	.463
Renal function				
Urea nitrogen, mean (*SD*)	6.45 (3.98)	6.46 (4.06)	6.39 (2.83)	.951
eGFR, mean (*SD*)	93.55 (34.04)	93.64 (34.29)	92.53 (32.32)	.901
CKD, %	24 (12.8)	22 (12.8)	2 (12.5)	1.000
ESRD, %	6 (3.2)	6 (3.5)	0	1.000
Cardiac biomarkers				
Elevated troponint, %	66 (35.1)	56 (32.6)	10 (62.5)	.016
Elevated myoglobin, %	123 (65.4)	114 (66.3)	9 (56.3)	.420
Elevated CK‐MB, %	6 (3.2)	6 (3.5)	0	1.000
Serum ions				
Sodium, mean (*SD*)	138.35 (5.99)	138.32 (6.08)	139.15 (3.88)	.851
Potassium, mean (*SD*)	3.53 (0.47)	3.54 (0.48)	3.35 (0.63)	.584
Phosphorous, mean (*SD*)	0.91 (0.43)	0.91 (0.44)	0.89 (0.01)	.949
Calcium, mean (*SD*)	2.19 (0.32)	2.19 (0.33)	2.19 (0.98)	.979
Magnesium, mean (*SD*)	0.83 (0.16)	0.83 (0.17)	0.79 (0.07)	.734
Hematoma volume, mean (*SD*)	30.04 (26.39)	26.31 (22.15)	64.18 (36.95)	.002
Change in hematoma volume from diagnostic to follow up CT, mean (*SD*)	2.20 (11.42)	5.56 (18.60)	1.95 (10.86)	.451
Hematoma site				
Lobe, %	64 (34)	60 (34.9)	4 (25)	.425
Deep, %	75 (39.9)	69 (40.1)	6 (37.5)	.838
Posterior fossa, %	26 (13.8)	24 (14)	2 (12.5)	1.000
Primary intraventricular hemorrhage, %	11 (5.9)	10 (5.8)	1 (6.3)	1.000
Multiple/undefined, %	12 (6.4)	9 (5.2)	3 (18.8)	.114
Intraventricular hemorrhage, %	84 (44.7)	76 (44.2)	8 (50)	.655
Combined with subarachnoid hemorrhage	27 (14.4)	26 (15.1)	1 (6.3)	.552
Model 1 OR (95% confidence interval)^a^		1	3.28 (1.08–9.96)	.037
Model 2 OR (95% confidence interval)^b^		1	5.19 (1.08–24.93)	.040

*SD*, standard deviation; IQR, interquartile range; GCS, the Glasgow Coma Scale; NIHSS, the National Institutes of Health Stroke Scale; PT, prothrombin time; APTT, activated partial thromboplastin time; INR, international normalized ratio; eGFR, estimated glomerular filtration rate; CKD, chronic kidney disease; ESRD, end stage renal disease; CK‐MB, creatine kinase‐MB mass.

a Adjusted for age, sex, GCS and NIHSS.

b Adjusted for additional history of hypertension, diabetes mellitus, cardiac events, previous stroke, smoking, systolic blood pressure on admission, chronic kidney disease and hematoma volume >30 ml.

After adjusting for age, sex, GCS and NIHSS, those with cerebral herniation were more likely to have elevated troponin (odds ratio [OR] 3.28, 95% confidence interval [CI], 1.08–9.96, *p* = .037). After adjusting for additional confounders, elevated serum troponin was still associated with the presence of cerebral herniation (OR, 5.19; 95% CI, 1.08–24.93, *p* = .040).

Next, the association between elevated troponin and the location of hematoma, as well as the etiology and the severity of ICH, was analyzed (Table 2). Patients with elevated troponin were older (*p* = .003), more likely to have diabetes mellitus (*p* = .016), had higher systolic blood pressure and diastolic blood pressure (*p* = .004, *p* = .013, respectively). As for the hematoma site, patients with elevated troponin had a higher proportion of hematoma in deep compared with those with normal troponin (50% vs. 34.4%, *p* = .037), and were more likely to be accompanied by hypertensive ICH (*p* < .001), whereas normal troponin was more likely to be accompanied by secondary ICH such as arteriovenous malformation (*p* = .034). In addition, patients with elevated troponin had lower GCS score and higher NIHSS score (*p* = .026, .005, respectively). And in‐hospital mortality was also higher among elevated troponin group compared with normal troponin group (16.7% vs. 4.9%, *p* = .007). After adjusting for only age and sex, elevated troponin was significantly associated with GCS < 8, as well as NIHSS > 10, whereas the relationship of elevated troponin with in‐hospital mortality and deep location of ICH became no longer significant (Table 3). After adjusting for additional confounders such as history of hypertension, diabetes mellitus, smoking, previous stroke and systolic blood pressure, elevated troponin was still associated with GCS < 8 (adjusted OR 2.34; 95% CI, 1.17–4.68, *p* = .016) and NIHSS > 10 (adjusted OR 2.06; 95% CI, 1.06–4.01, *p* = .033).

**Table 2:** Demographic and clinical data by presence of elevated troponin

	Elevated troponin (*n* = 66)	Normal troponin (*n* = 122)	*p* Value
Age, mean (*SD*)	61.17 (17.12)	53.72 (16.08)	.003
Male, %	45 (68.2)	69 (56.6)	.119
Hypertension, %	36 (54.5)	51 (41.8)	.094
Diabetes mellitus, %	10 (15.2)	6 (4.9)	.016
Cardiac events, %	5 (7.6)	9 (7.4)	1.000
Previous stroke, %	8 (12.1)	13 (10.7)	.761
Alcohol intake, %	7 (10.6)	22 (18)	.178
Smoking, %	17 (25.8)	32 (26.2)	.944
Systolic blood pressure, mean (*SD*)	170 (36.76)	155.03 (31.36)	.004
Diastolic blood pressure, mean (*SD*)	100.26 (23.88)	91.65 (18.56)	.013
CKD	20 (30.3)	4 (3.3)	<.001
ESRD	6 (9.1)	0	.003
Hematoma volume at admission, mean (*SD*)	35.10 (29.41)	26.85 (23.91)	.084
Hematoma site			
Lobe, %	13 (19.7)	51 (41.8)	.002
Deep, %	33 (50)	42 (34.4)	.037
Posterior fossa, %	8 (12.1)	18 (14.8)	.618
Primary intraventricular hemorrhage, %	6 (9.1)	5 (4.1)	.286
Multiple/undefined, %	6 (9.1)	6 (4.9)	.421
IVH, %	36 (54.5)	48 (39.3)	.045
Etiology			.005
Hypertension, %	53 (80.3)	66 (54.1)	<.001
Aneurysm, %	5 (7.6)	18 (14.8)	.152
Arteriovenous malformation, %	2 (3.0)	15 (12.3)	.034
Moyamoya disease, %	3 (4.5)	1 (0.8)	.246
Severe systemic disease	0	5 (4.1)	.164
Unknown	6 (9.1)	19 (15.6)	.211
Severity of stroke			
GCS, median (IQR)	10 (7.5)	13 (8.25)	.026
NIHSS, median (IQR)	12 (20)	8.5 (16.25)	.005
In‐hospital mortality, %	11 (16.7)	6 (4.9)	.007

*SD*, standard deviation; IQR, interquartile range; GCS, the Glasgow Coma Scale; NIHSS, the National Institutes of Health Stroke Scale; CKD, chronic kidney disease; ESRD, end stage renal disease; IVH, intraventricular hemorrhage.

**Table 3:** Multivariate analysis for GCS score, NIHSS Score and in‐hospital mortality

Outcome	Category	Adjusted OR (1)	Adjusted 95% CI (1)	*p* Value	Adjusted OR (2)	Adjusted 95% CI (2)	*p* Value
GCS<8	Elevated troponin	2.60	1.34–5.05	.005	2.34	1.17–4.68	.016
	Normal troponin	Reference	—		Reference	—	
NIHSS > 10	Elevated troponin	2.34	1.24–4.41	.008	2.06	1.06–4.01	.033
	Normal troponin	Reference	—		Reference	—	
In‐hospital mortality	Elevated troponin	2.63	0.87–7.93	.085	2.24	0.69–7.28	.179
	Normal troponin	Reference	—		Reference	—	
Deep location of ICH	Elevated troponin	1.64	0.87–3.08	.126	1.32	0.67–2.61	.424
	Normal troponin	Reference	—		Reference	—	

Adjusted (1): adjusted for age and sex. Adjusted (2): adjusted for age, sex, history of hypertension, diabetes mellitus, smoking, previous stroke and systolic blood pressure.

**Figure 1:**
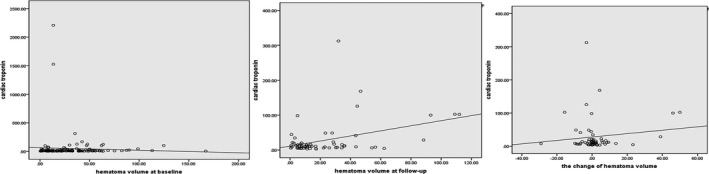
The variation of troponin along with hematoma volume at baseline and follow‐up in the scatter plot

**Figure 2:**
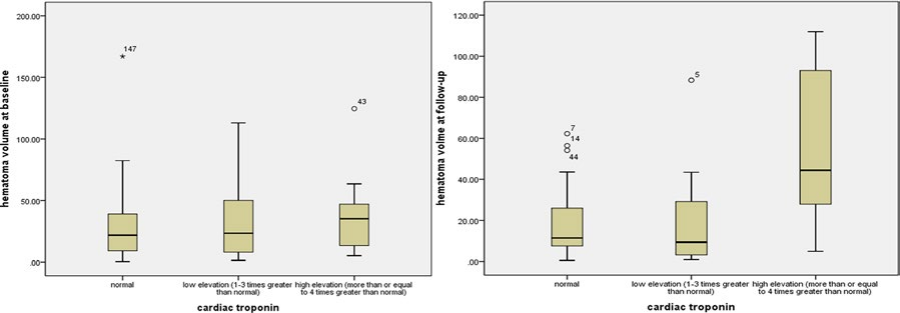
The variation of troponin along with hematoma volume at baseline and follow‐up in the box diagram

As for hematoma volume, those with elevated troponin had large hematoma volume at follow‐up in bivariate correlation (correlation coefficient, .375, *p* = .003) and linear regression (β, 0.370, 95% CI, 0.062–0.320, *p* = .005) than those with normal troponin. However, elevated troponin was not associated with hematoma volume at admission and the change in hematoma volume from diagnostic to follow up CT. Figures 1 and 2 showed the variation of troponin along with hematoma volume at baseline and follow‐up in the scatter plot and box diagram.

## 4 Discussion

Cerebral herniation is a rare and devastating complication in ICH patients. Intracerebral hematoma could lead to the rapid increase of cerebral pressure and result in the downward displacement of brain tissue through the notch (Wu et al., 2014). To date, the main reports of cerebral herniation consist mostly of clinical reports (Rehman, Ali, Tawil, & Yonas, 2008; Sampaio, Dias da Costa, Rocha, & Leao, 2016), and information about herniation after ICH reported in the literature is limited.

In this retrospective cohort study, we found that elevated troponin was not just associated with the frequency of cerebral herniation, but also large hematoma volume at follow‐up, suggesting that serum troponin levels played a pivotal role in ICH patients and are associated with the severity of stroke. Furthermore, we observed that ICH patients with elevated troponin were more likely to have a higher systolic blood pressure levels, a higher frequency of CKD and ESRD and a more frequent deep location, possibly shared the pathophysiology of cerebral herniation. However, the change in hematoma volume from diagnostic to follow up CT between patients with and without elevated troponin, as well as between those with and without cerebral herniation, was not significant. Previous study reports that approximately one‐third of ICH patients had significant change in hematoma volume (Brott et al., 1997), suggesting that not all patients had the risk of change in hematoma volume or hematoma expansion. The CT spot sign, hematoma morphologic appearance (Blacquiere et al., 2015), and the etiologic subtype (Cappellari et al., 2015) also had great influence on change in hematoma volume.

The mechanisms that can explain the association of elevated troponin with high frequency of brain herniation are complex and incompletely understood. First, Cardiac troponin is a highly sensitive and specific marker of myocardial injury (Agewall, Giannitsis, Jernberg, & Katus, 2011; Thygesen, Alpert, & White, 2007). The rapid increase of intracranial pressure, especially in patients with cerebral herniation, can induce the fast‐releasing catecholamines as a result of sympathoadrenal activation which act on myocardial cell (Hamann et al., 1995; Leow, Loh, Kiat Kwek, & Ng, 2007). The high concentration of catecholamines in myocardial cell would cause a calcium abnormality (Mann, Kent, Parsons, & Cooper, 1992) and bring about a reduction of contractility in the myocardium. Second, elevated cardiac troponin was associated with large hematoma volume at follow‐up in bivariate correlation and linear regression, which adjusted potential confounders such as age, GCS score and systolic blood pressure at admission, than those with normal troponin in our study. In addition, ICH patients with elevated troponin had a higher systolic blood pressure and diastolic blood pressure. To our knowledge, high blood pressure may promote on‐going bleeding in brain and even caused hematoma expansion, and promoted the formation of encephaledema through hydrostatic or oncotic pressure gradients (Chen, Chen, Hsu, & Hogan, 1989; Sykora et al., 2008). Third, elevated troponin may reflect poor renal function. In the present study, elevated troponin was associated with high frequency of CKD and ESRD. The results were in accordance with recent studies about the relationship of troponin and renal function (Teo, 2015). Furthermore, renal dysfunction had a 2.3‐fold higher hematoma volume compared to those with normal renal function (Molshatzki et al., 2011). And these findings indicated another mechanism for large hematoma volume and high frequency of cerebral herniation.

Our study indicated that ICH patients with elevated troponin had a higher proportion of in‐hospital mortality. Whereas the relationship of elevated troponin to in‐hospital mortality became no longer significant after adjusting confounding factors.

Limitations of this study include the single‐center design and retrospective analysis with exclusion of those subjects without the data of serum troponin. Therefore, there might be selection bias in this study. Other limitations were small sample size for multivariable analysis, single measurement of troponin and the possible existence of the unmeasured confounding factors which may explain some of our findings. Future prospectively large studies are warranted to make this distinction.

## 5 Conclusions

Elevated troponin, even after adjusting potential confounders, is associated with an almost five‐fold increased risk of cerebral herniation. The possibility of cerebral herniation should be considered when ICH patients with large hematoma volume and elevated troponin.

## Conflicts of Interest

None declared.

## References

[brb3697-bib-0001] Agewall, S. , Giannitsis, E. , Jernberg, T. , & Katus, H. (2011). Troponin elevation in coronary vs. non‐coronary disease. European Heart Journal, 32(4), 404–411. 21169615 10.1093/eurheartj/ehq456

[brb3697-bib-0002] Batal, O. , Jentzer, J. , Balaney, B. , Kolia, N. , Hickey, G. , Dardari, Z. , … Schmidhofer, M. (2016). The prognostic significance of troponin I elevation in acute ischemic stroke. Journal of Critical Care, 31(1), 41–47. 26547807 10.1016/j.jcrc.2015.09.018

[brb3697-bib-0003] Blacquiere, D. , Demchuk, A. M. , Al‐Hazzaa, M. , Deshpande, A. , Petrcich, W. , Aviv, R. I. , … PREDICT/Sunnybrook ICH CTA Study Group (2015). Intracerebral hematoma morphologic appearance on noncontrast computed tomography predicts significant hematoma expansion. Stroke, 46(11), 3111–3116. 26451019 10.1161/STROKEAHA.115.010566

[brb3697-bib-0004] Bor‐Seng‐Shu, E. , Paiva, W. S. , Figueiredo, E. G. , Fujimoto, Y. , de Andrade, A. F. , Fonoff, E. T. , & Teixeira, M. J. (2013). Posttraumatic refractory intracranial hypertension and brain herniation syndrome: Cerebral hemodynamic assessment before decompressive craniectomy. BioMed Research International, 2013, 750809. 24377095 10.1155/2013/750809 PMC3860083

[brb3697-bib-0005] Brott, T. , Adams Jr, H. P. , Olinger, C. P. , Marler, J. R. , Barsan, W. G. , Biller, J. , … Walker, M. (1989). Measurements of acute cerebral infarction: A clinical examination scale. Stroke, 20(7), 864–870. 2749846 10.1161/01.str.20.7.864

[brb3697-bib-0006] Brott, T. , Broderick, J. , Kothari, R. , Barsan, W. , Tomsick, T. , Sauerbeck, L. , … Khoury, J. (1997). Early hemorrhage growth in patients with intracerebral hemorrhage. Stroke, 28(1), 1–5. 8996478 10.1161/01.str.28.1.1

[brb3697-bib-0007] Cappellari, M. , Zivelonghi, C. , Moretto, G. , Micheletti, N. , Carletti, M. , Tomelleri, G. , & Bovi, P. (2015). The etiologic subtype of intracerebral hemorrhage may influence the risk of significant hematoma expansion. Journal of the Neurological Sciences, 359(1–2), 293–297. 26671130 10.1016/j.jns.2015.11.024

[brb3697-bib-0008] Chen, S. T. , Chen, S. D. , Hsu, C. Y. , & Hogan, E. L. (1989). Progression of hypertensive intracerebral hemorrhage. Neurology, 39(11), 1509–1514. 2812332 10.1212/wnl.39.11.1509

[brb3697-bib-0009] Everett, B. M. , Zeller, T. , Glynn, R. J. , Ridker, P. M. , & Blankenberg, S. (2015). High‐sensitivity cardiac troponin I and B‐type natriuretic Peptide as predictors of vascular events in primary prevention: Impact of statin therapy. Circulation, 131(21), 1851–1860. 25825410 10.1161/CIRCULATIONAHA.114.014522 PMC4444427

[brb3697-bib-0010] Gower, D. J. , Baker, A. L. , Bell, W. O. , & Ball, M. R. (1987). Contraindications to lumbar puncture as defined by computed cranial tomography. Journal of Neurology, Neurosurgery, and Psychiatry, 50(8), 1071–1074. 10.1136/jnnp.50.8.1071 PMC1032242 3655817

[brb3697-bib-0011] Hamann, G. F. , Strittmatter, M. , Hoffmann, K. H. , Holzer, G. , Stoll, M. , Keshevar, T. , … Schimrigk, K. (1995). Pattern of elevation of urine catecholamines in intracerebral haemorrhage. Acta Neurochirurgica, 132(1–3), 42–47. 7754857 10.1007/BF01404846

[brb3697-bib-0012] Koenig, M. A. , Bryan, M. , Lewin III, J. L. , Mirski, M. A. , Geocadin, R. G. , & Stevens, R. D. (2008). Reversal of transtentorial herniation with hypertonic saline. Neurology, 70(13), 1023–1029. 18272864 10.1212/01.wnl.0000304042.05557.60

[brb3697-bib-0013] Kothari, R. U. , Brott, T. , Broderick, J. P. , Barsan, W. G. , Sauerbeck, L. R. , Zuccarello, M. , & Khoury, J. (1996). The ABCs of measuring intracerebral hemorrhage volumes. Stroke, 27(8), 1304–1305. 8711791 10.1161/01.str.27.8.1304

[brb3697-bib-0014] Lei, C. , Wu, B. , Liu, M. , Cao, T. , Wang, Q. , Dong, W. , & Chang, X. (2015). VSARICHS: A simple grading scale for vascular structural abnormality‐related intracerebral haemorrhage. Journal of Neurology, Neurosurgery, and Psychiatry, 86(8), 911–916. 10.1136/jnnp-2014-308777 25280916

[brb3697-bib-0015] Leow, M. K. , & Loh, K. C. , Kiat Kwek, T. , & Ng, P. Y. (2007). Catecholamine and metanephrine excess in intracerebral haemorrhage: Revisiting an obscure yet common “pseudophaeochromocytoma”. Journal of Clinical Pathology, 60(5), 583–584. 17513524 10.1136/jcp.2006.036640 PMC1994550

[brb3697-bib-0016] Mann, D. L. , Kent, R. L. , Parsons, B. , & Cooper, G. (1992). Adrenergic effects on the biology of the adult mammalian cardiocyte. Circulation, 85(2), 790–804. 1370925 10.1161/01.cir.85.2.790

[brb3697-bib-0017] Molshatzki, N. , Orion, D. , Tsabari, R. , Schwammenthal, Y. , Merzeliak, O. , Toashi, M. , & Tanne, D. (2011). Chronic kidney disease in patients with acute intracerebral hemorrhage: Association with large hematoma volume and poor outcome. Cerebrovascular Diseases, 31(3), 271–277. 21178352 10.1159/000322155

[brb3697-bib-0018] Naidech, A. M. , Kreiter, K. T. , Janjua, N. , Ostapkovich, N. D. , Parra, A. , Commichau, C. , … Mayer, S. A. (2005). Cardiac troponin elevation, cardiovascular morbidity, and outcome after subarachnoid hemorrhage. Circulation, 112(18), 2851–2856. 16267258 10.1161/CIRCULATIONAHA.105.533620

[brb3697-bib-0019] National Kidney Foundation (2002). K/DOQI clinical practice guidelines for chronic kidney disease: Evaluation, classification, and stratification. American Journal of Kidney Diseases, 39(2 Suppl. 1), S1–S266. 11904577

[brb3697-bib-0020] Raza, F. , & Alkhouli, M. (2014). Elevated cardiac troponin in acute stroke without acute coronary syndrome predicts long‐term adverse cardiovascular outcomes. Stroke Research and Treatment, 2014, 621650. 25530906 10.1155/2014/621650 PMC4235111

[brb3697-bib-0021] Rehman, T. , Ali, R. , Tawil, I. , & Yonas, H. (2008). Rapid progression of traumatic bifrontal contusions to transtentorial herniation: A case report. Cases Journal, 1(1), 203. 18831756 10.1186/1757-1626-1-203 PMC2566562

[brb3697-bib-0022] Sampaio, L. , Dias da Costa, J. M. , Rocha, R. , & Leao, M. (2016). Brain herniation into the dural venous sinus. The Journal of Pediatrics, 174, 276. 27174142 10.1016/j.jpeds.2016.04.010

[brb3697-bib-0023] Stevens, R. D. , Shoykhet, M. , & Cadena, R. (2015). Emergency neurological life support: Intracranial hypertension and herniation. Neurocritical Care, 23(Suppl. 2), 76–82. 10.1007/s12028-015-0168-z PMC4791176 26438459

[brb3697-bib-0024] Sykora, M. , Diedler, J. , Rupp, A. , Turcani, P. , Rocco, A. , & Steiner, T. (2008). Impaired baroreflex sensitivity predicts outcome of acute intracerebral hemorrhage. Critical Care Medicine, 36(11), 3074–3079. 18824914 10.1097/CCM.0b013e31818b306d

[brb3697-bib-0025] Teo, B. W. (2015). Serum high‐sensitivity troponin concentrations in a multi‐ethnic Asian population of stable chronic kidney disease patients. Clinical Chemistry and Laboratory Medicine, 53(5), e121–e123. 25490031 10.1515/cclm-2014-0862

[brb3697-bib-0026] Thalin, C. , Rudberg, A. S. , Johansson, F. , Jonsson, F. , Laska, A. C. , Nygren, A. T. , … Aspberg, S. (2015). Elevated troponin levels in acute stroke patients predict long‐term mortality. Journal of Stroke and Cerebrovascular Diseases, 24(10), 2390–2396. 26236002 10.1016/j.jstrokecerebrovasdis.2015.06.043

[brb3697-bib-0027] Thygesen, K. , Alpert, J. S. , & White, H. D. (2007). Universal definition of myocardial infarction. European Heart Journal, 28(20), 2525–2538. 17951287 10.1093/eurheartj/ehm355

[brb3697-bib-0028] Wu, H. , Yang, S. F. , Qiu, Y. M. , Dai, J. , Li, S. Q. , Zhang, X. H. , & Miao, Y. F. (2014). The diagnosis and surgical treatment of central brain herniations caused by traumatic bifrontal contusions. Journal of Craniofacial Surgery, 25(6), 2105–2108. 25304144 10.1097/SCS.0000000000001050

[brb3697-bib-0029] Zhang, L. , Wang, Z. , & Qi, S. (2015). Cardiac troponin elevation and outcome after subarachnoid hemorrhage: A systematic review and meta‐analysis. Journal of Stroke and Cerebrovascular Diseases, 24(10), 2375–2384. 26227321 10.1016/j.jstrokecerebrovasdis.2015.06.030

